# Substrate‐specific mitochondrial dysfunction and metabolomic profiles in type 2 diabetic rat hearts

**DOI:** 10.1113/EP094055

**Published:** 2026-06-19

**Authors:** Toan Pham

**Affiliations:** ^1^ Auckland Bioengineering Institute The University of Auckland Auckland New Zealand; ^2^ Department of Engineering Science and Biomedical Engineering University of Auckland Auckland New Zealand

**Keywords:** adenosine triphosphate, cardiac mitochondria, metabolite, mitochondrial respiration, reactive oxygen species, type 2 diabetes

## Abstract

Type 2 diabetes (T2D) greatly alters cardiac fuel handling, yet how mitochondrial function adapts to the diabetic substrate environment remains unclear. This study investigated substrate‐specific cardiac mitochondrial bioenergetics from a T2D rat model induced by a high‐fat diet and low‐dose streptozotocin. High‐resolution respirometry and fluorimetry were used to measure mitochondrial O_2_ flux, ATP flux, reactive oxygen species production rate and mitochondrial membrane potential in cardiac tissue homogenates in two key substrate conditions: carbohydrate and fatty acid. Liquid chromatography–mass spectrometry was used to analyse the abundance of metabolites involved in the Krebs cycle and in various fatty acid metabolic pathways. Carbohydrate‐supported mitochondrial respiration, ATP flux, reactive oxygen species production and mitochondrial membrane potential were preserved in T2D myocardium. In contrast, fatty acid‐supported mitochondrial respiration and ATP flux in oxidative phosphorylation were significantly decreased despite increased myocardial abundance of several fatty acid species, including palmitoleic acid, *cis*‐8‐heptadecenoic acid and linoleic acid. Metabolite intermediates of the Krebs cycle were largely unchanged. These findings reveal a substrate‐specific energetic defect in the diabetic heart, in which excess fatty acid supply is not matched by mitochondrial oxidative capacity, leading to metabolic inflexibility and impaired ATP generation. This work provides mechanistic insight into how nutrient overload contributes to mitochondrial inefficiency in T2D and establishes a foundation for future studies targeting lipid–mitochondria interactions.

## Abstract (highlights)

- **What is the central question of this study?**
  How do mitochondrial energetics and metabolomic profiles alter in type 2 diabetic rat hearts?
- **What is the main finding and its importance?**
  By combining high‐resolution respirometry and metabolomics in a well‐established diabetes‐like rat model, this study shows that carbohydrate‐supported mitochondrial function remains preserved. In contrast, fatty acid‐supported mitochondrial function is impaired, despite higher fatty acid abundance in the diabetic heart. This study provides the first merged evidence linking substrate overloading to substrate‐specific mitochondrial dysfunction in the diabetic heart, offering a foundation for future therapeutic research.

## 1 INTRODUCTION

Type 2 diabetes (T2D) is a common chronic disease, characterised by persistently high blood glucose levels and a series of other metabolic disturbances. T2D is associated with a range of microvascular and macrovascular complications that significantly increase the risk of dysfunction across multiple organ systems. Cardiovascular disease remains the leading cause of morbidity and mortality in diabetic patients, and heart failure accounts for nearly 80% of all deaths (Boudina & Abel, 2007). Diabetic cardiomyopathy is characterised by left ventricular hypertrophy, altered substrate metabolism (Amaral & Okonko, 2015; Sharma et al., 2004; Stanley et al., 1997) and, eventually, decreased contractile function (Montaigne et al., 2014). A better understanding of the underlying processes of T2D‐induced heart dysfunction is crucial for establishing effective therapeutic options.

The heart relies on a continuous high‐energy supply from mitochondria to sustain high contractile workloads. Mitochondria occupy approximately one‐third of cardiomyocyte volume and generate >95 % of ATP through oxidative phosphorylation (OXPHOS) (Barth et al., 1992). Evidence suggests that mitochondrial dysfunction might precede cardiac contractile dysfunction and could be a key contributor to the decreased myocardial contractility seen in T2D patients (Montaigne et al., 2014) and in animal models of diabetes (Li et al., 2022; Marciniak et al., 2014; Tang et al., 2023). Human diabetic hearts consistently show decreased mitochondrial respiration supported by fatty acid substrates (Anderson et al., 2011; Montaigne et al., 2014), whereas carbohydrate‐supported mitochondrial respiration is often preserved (Anderson et al., 2011; Ljubkovic et al., 2019; Montaigne et al., 2014). Paradoxically, T2D is characterised by elevated circulating free fatty acids and triglycerides in diabetic patients (Anderson et al., 2009) and rats (Tang et al., 2023), increased myocardial fatty uptake and lipid accumulation (Bonen et al., 2009; Chabowski et al., 2008; Finck et al., 2002; Ljubkovic et al., 2019) and a metabolic shift towards greater reliance on fatty acid oxidation (Cortassa et al., 2020). This pattern suggests that mitochondrial dysfunction in diabetes is not global but substrate dependent, potentially reflecting a mismatch between nutrient supply and mitochondrial oxidative capacity. However, the mechanistic basis for this mismatch remains unresolved.

To address this gap, I combined high‐resolution respirometry, fluorometry and targeted mass spectrometry‐based metabolomics in myocardial tissues from a T2D rat model. This integrated approach allowed simultaneous measurements of mitochondrial respiration, H_2_O_2_ emission, ATP production and mitochondrial membrane potential in both carbohydrate‐ and fatty acid‐supported conditions, while assessing the abundance of myocardial metabolites in T2D hearts. I hypothesised that chronic substrate excess in T2D would selectively impair fatty acid‐supported mitochondrial function. The findings from this study reveal that the fatty acid substrate–mitochondria mismatch provides mechanistic insight into metabolic inflexibility in T2D and highlight lipid overload as a driver of mitochondrial inefficiency.

## 2 MATERIALS AND METHODS

### 2.1 Ethical approval

All animal handling procedures were conducted in accordance with protocols approved by the University of Auckland Animal Ethics Committee (AEC22653). Male Wistar rats (150–200 g) were obtained from the Vernon Jansen Unit animal facility at the University of Auckland. T2D was induced using a combination of high‐fat diet feeding and low‐dose streptozotocin (STZ) injection. Rats assigned to the T2D group received a high‐fat diet (43% digestible energy from lipids; SF04‐001, Specialty Feeds, Australia) for 15 weeks. At week 8, these animals received an intraperitoneal injection of STZ (25 mg/kg in citrate buffer pH 4.5). Control rats received standard chow and an injection of citrate buffer only. All rats were housed under a 12 h–12 h light–dark cycle with ad libitum access to food and water. Body weight and blood glucose were monitored weekly. Animals that developed only mild hyperglycaemia (8–10 mmol/L), consistent with early‐stage or prediabetic phenotypes, were excluded to ensure a uniform late‐stage T2D cohort. At week 15, rats were transported to the laboratory and acclimated for ≥1 h in a climate‐controlled chamber to minimise transport‐related stress before experimentation.

### 2.2 Tissue collection

On each experimental day, rats were deeply anaesthetised by isoflurane inhalation (5% in O_2_) and received a subcutaneous injection of heparin (1000 IU/kg). Following cervical dislocation, the heart was rapidly excised and perfused in Langendorff mode with oxygenated Tyrode solution at room temperature to remove residual blood in the coronary circulation. The Tyrode solution contained (in millimoles per litre): 130 NaCl, 6 KCl, 1 MgCl_2_, 0.3 CaCl_2_, 0.5 NaH_2_PO_4_, 10 HEPES, 10 glucose and 20 2,3‐butanedione monoxime (pH 7.4, adjusted with Tris). Under a dissecting microscope, the heart was dissected to open both ventricles for thickness measurement. Left and right ventricular free wall thicknesses were measured using a dissecting microscope graticule. Heart mass and tibial length were also measured. Blood samples were collected from the opened thoracic cavity into an EDTA blood tube immediately after heart excision, then centrifuged at 2000*g* for 10 min at 4°C. Plasma samples were snap‐frozen within 10 min of collection and stored at −80°C for later metabolomic analyses.

### 2.3 Tissue homogenisation for mitochondrial function assays

Fresh left ventricular tissues were used for mitochondrial function assays. Approximately 30 mg of tissue was blotted, weighed, and transferred into 750 µL of cold modified MiRO5 buffer. The MiRO5 buffer contained (in millimoles per litre): 110 sucrose, 60 potassium lactobionate, 20 HEPES, 20 taurine, 10 KH_2_PO_4_, 0.5 EGTA, 1 g/L bovine serum albumin fatty acid free (pH 7.1 adjusted at 30°C).

Heart samples were carefully minced with scissors, then homogenised for 10 s at medium speed using a tissue homogeniser (Omni International, Hennesaw, GA, USA). Homogenates were kept on ice and used immediately for mitochondrial respirometry and fluorometry. The remaining homogenates and left ventricular tissues were stored at −80°C for later enzymatic and metabolomic analyses.

### 2.4 High‐resolution respirometry and fluorimetry measurements

Mitochondrial respiration, ATP production, mitochondrial membrane potential and H_2_O_2_ emission were assessed using an O2k high‐resolution fluorespirometer (O2k, Oroboros Instruments, Innsbruck, Austria). The O2k device has two chambers, each containing a polarographic oxygen sensor and a stopper, allowing sequential titration of substrates, uncouplers and inhibitors. A detachable fluorimeter (Oroboros Instruments) was positioned at each chamber window to enable simultaneous measurements of fluorescence and O_2_ flux. The assay medium O_2_ concentration was calibrated to 195 µmol/L at barometric pressure, and all measurements were performed at 37°C. The O_2_ concentration was maintained between 100 and 195 µmol/L throughout the experiments to maintain comparable O_2_ conditions, except during hypoxic conditions in titration protocol 2. Substrates, uncouplers and inhibitors were added sequentially using Hamilton syringes. Two titration protocols were performed in MiRO5 buffer.

#### 2.4.1 Mitochondrial H_2_O_2_ production rate and O_2_ flux using titration protocol 1

Net mitochondrial H_2_O_2_ production was measured simultaneously with O_2_ flux using Amplex UltraRed (AUR, A36006, Thermo Fisher Scientific). AUR (25 µmol/L), horseradish peroxidase (10 U) and superoxide dismutase (10 U) were added to each chamber. Fluorescence (excitation 525 nm/emission 550 nm) was calibrated using three titrations of H_2_O_2_ (0.122 µmol/L) and allowed to equilibrate before adding samples to the chamber (Broome et al., 2022; Fang et al., 2026). Measurements were performed in carbohydrate‐supported conditions, as in titration protocol 1.

#### 2.4.2 Mitochondrial membrane potential and O_2_ flux using titration protocol 1

Mitochondrial membrane potential was measured using safranine (Sigma Aldrich) fluorescence at excitation and emission wavelengths of 495 and 587 nm, respectively. Mitochondrial depolarisation causes the redistribution of a high concentration of quenched safranine from mitochondria to the cytosol, where the lower concentration results in dequenching and an increase in fluorescence (Goo et al., 2013; Power et al., 2014). Safranine (2 µmol/L) was added to each chamber, followed by calibration of the fluorescence signal. Measurements were performed in carbohydrate‐supported conditions, as in titration protocol 1.

#### 2.4.3 Mitochondrial ATP production rate and O_2_ flux using titration protocols 1 and 2

ATP production rate was measured using Magnesium Green (MgG, pentapotassium salt, cell impermeant, M3733, Thermo Fisher Scientific) fluorescence at excitation and emission wavelengths of 470 and 520 nm, respectively (Fang et al., 2026; Power et al., 2014). MgG (5 µmol/L) was added to the medium, and MgCl_2_ (1 mmol/L) was added to each chamber to calibrate the MgG signal. Measurements were performed in both carbohydrate‐ and fatty acid‐supported conditions in separate experimental runs.

#### 2.4.4 Titration protocol 1: Carbohydrate‐supported respiration

Tissue homogenate (2 mg) and MgCl_2_ (1 mmol/L) were added to each chamber and allowed to equilibrate. NADH‐linked leak respiration was determined using malate (2 mmol/L), glutamate (10 mmol/L) and pyruvate (5 mmol/L). Succinate (10 mmol/L) was added to assess NADH‐ and complex II (CII)‐linked leak respiration; despite the presence of a small amount of ADP (∼0.03 µmol/L) in homogenate samples, it might contribute to negligible OXPHOS respiration. Excess ADP (2.5 mmol/L) was added to stimulate OXPHOS respiration. The uncoupler carbonyl cyanide *m*‐chlorophenyl hydrazone (CCCP, 0.5 µmol/L) was used to induce uncoupled respiration as a measure of maximal electron transport system capacity. Antimycin A (1 µmol/L) was added to inhibit complex III to determine any residual O_2_ flux. Complex IV (CIV) respiration was measured using ascorbate (2 mmol/L) and *N*,*N*,*N*′,*N*′‐tetramethyl‐*p*‐phenylenediamine dihydrochloride (TMPD, 0.5 mmol/L), followed by inhibition with azide (100 mmol/L).

#### 2.4.5 Titration protocol 2: Fatty acid‐supported respiration

To assess the impact of fatty acid metabolism on mitochondrial respiration, a low concentration of malate (0.1 mmol/L) and palmitoyl‐l‐carnitine (40 µmol/L) were added to initiate leak respiration. ADP (2.5 mmol/L) stimulated fatty acid‐mediated OXPHOS respiration, followed by succinate (10 mmol/L) for CII‐mediated OXPHOS. The tissue was then allowed to consume all oxygen in the measurement chamber, and the O_2_ concentration reached zero. Tissue was maintained in this anoxic state for 10–15 min to mimic O_2_ depletion in vivo during ischaemic conditions. The O_2_ concentration was then restored to normal by opening the stoppers to allow air equilibration; this approach allowed me to assess the anoxia–reoxygenation sequence on mitochondrial function after a long‐term diabetic insult. Oligomycin (2 µmol/L), an ATP synthase inhibitor, was added to stop mitochondrial ATP production. Antimycin A (1 µmol/L) was added to inhibit complex III to determine any residual O_2_ flux. Because cytochrome *c* was not added, outer mitochondrial membrane integrity could not be assessed; this limitation is acknowledged in the Discussion.

### 2.5 Citrate synthase activity

A protease inhibitor cocktail (25 µL, cOmplete Mini EDTA‐free, Roche, Mannheim, Germany) was added to frozen homogenate samples. The samples were thawed on ice, vortexed, and centrifuged at 5000*g* for 10 min at 4°C. The supernatants were diluted 25‐fold in PBS and assayed for citrate synthase (CS) activity. Absorbance was measured at 412 nm every 30 s for a duration of 10 min using a microplate reader at room temperature in Tris–HCl buffer (50 mmol/L, pH 8) containing oxaloacetate (0.5 mmol/L), acetyl‐CoA (0.1 mmol/L) and 5,5‐dithiobis‐(2‐nitrobenzoic acid) (0.2 mmol/L). CS activity was determined from the absorbance slope using an extinction coefficient value of 13 600 L/mol/cm and normalised for protein content (Bicinchoninic acid (BCA) protein assay kit, Thermo Fisher Scientific, Waltham, MA, USA).

### 2.6 Plasma biochemistry analysis

Plasma metabolite concentrations were measured on a Hitachi c311 autoanalyser (Hitachi High Technologies Corporation, Tokyo, Japan). Glucose, urea, alanine aminotransferase, albumin, lactate dehydrogenase, aspartate aminotransferase, creatinine, uric acid, and lipid profile markers (high‐density lipoprotein, low‐density lipoprotein and triglycerides) were measured using colourimetry assays. C‐Reactive protein was analysed by particle‐enhanced immunoturbidimetric assay (Roche Diagnostics, Mannheim, Germany).

### 2.7 Myocardial metabolomics

Frozen left ventricular tissues were sent to the Centre for Genomics, Proteomics and Metabolomics at the University of Auckland for metabolite profiling. Briefly, samples were freeze‐dried overnight, and 20 mg dry mass was processed for methyl chloroformate derivatisation and analysed using gas chromatography–mass spectrometry (GC7890B coupled to MSD5977C; Agilent Technologies). Extraction and derivatisation were performed according to established protocols (Smart et al., 2010). Metabolite abundances were normalised to dry tissue mass.

### 2.8 Data analyses

Respirometric and fluorometric data were recorded and analysed offline using DatLab v.7.1 (Oroboros Instruments). The O_2_ flux data were corrected for residual O_2_ flux following the addition of antimycin A, except for CIV respiration, for which O_2_ flux data derived from the autooxidation effect were subtracted for O_2_ flux after CIV inhibition with sodium azide. The H_2_O_2_ measurements were corrected for background H_2_O_2_ levels, which were measured before adding homogenate samples. ATP flux signals were calibrated using separate assays without tissue samples, as previously described (Pham et al., 2014), and corrected for the background signal attributable to ATP synthase inhibition by oligomycin. Data were normalised to either tissue wet mass or CS activity.

### 2.9 Statistical analyses

Statistical analyses and figure generations were performed with GraphPad Prism v.10.0 (GraphPad Software, San Diego, CA, USA). Data analysis included nine control rats and six T2D rats. A two‐way ANOVA was used to examine variables with two factors (group × respiratory state or blood glucose × time) across all metabolites and mitochondrial data, followed by Fisher's least significant difference *post hoc* test. Student's unpaired *t*‐tests were used for single‐variable comparisons. All data are presented as the mean ± SD, with significant difference declared at *P* < 0.05. Sample numbers for each experiment are reported in the figure legends.

## 3 RESULTS

### 3.1 Morphological characteristics of the T2D animals

**FIGURE 1:**
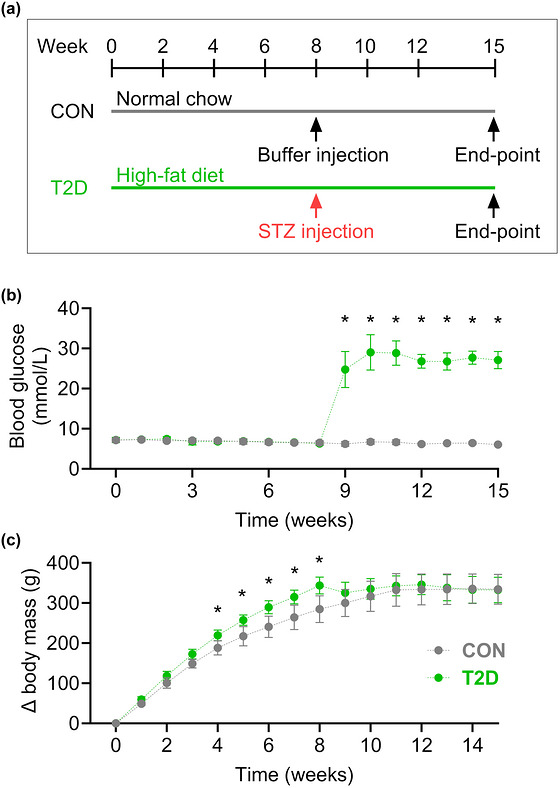
Weekly monitoring of animal phenotypes over 15 weeks. (a) Study time line. (b) Non‐fasting blood glucose. (c) Change in rat body mass. At week 8, the T2D group received an STZ injection, whereas the CON group received a buffer injection only. Following STZ injection, T2D rats developed marked hyperglycaemia and transient weight loss, after which body mass stabilised despite continued high‐fat feeding. CON group, *n* = 9, grey; T2D group, *n* = 6, green. Data are presented as the mean ± SD. Statistical analyses were performed using two‐way ANOVA with Fisher's least significant difference *post hoc* testing. ^*^ *P* < 0.05. Abbreviations: CON, control; STZ, streptozotocin; T2D, type 2 diabetes.

Figure 1 shows changes in blood glucose levels and body weight of all rats over 15 weeks. The T2D rats fed the high‐fat diet gained more weight than the control animals fed the standard chow diet after 4 weeks, while their blood glucose levels remained unchanged. Following an STZ injection at week 8, T2D rats exhibited increased blood glucose levels of >25 mmol/L. They developed initial body mass loss, with no subsequent weight gain despite continued high‐fat diet consumption, indicating an established or later stage of T2D. There was no significant difference in weight gain between groups after week 8 following the high‐fat diet.

Table 1 shows the morphological characteristics of the two rat groups. Compared with control animals, T2D rats exhibited increased heart mass‐to‐body mass ratio and left ventricular free wall thickness, indicating the development of cardiac hypertrophy. No significant differences between groups were detected in other parameters, including changes in body mass, heart mass, tibial length, right ventricular thickness and the heart mass‐to‐tibial length ratio.

**TABLE 1:** Morphological characteristics of the rats at the end of the experiment.

Parameter	CON (*n* = 9)	T2D (*n* = 6)	*P*‐value
Initial body mass, g	164.8 ± 5.5	148 ± 12.4	0.026*
Body mass, g	498.4 ± 39.7	471.9 ± 49.4	0.516
Change in body mass, g	333.7 ± 36.5	323 ± 45.9	0.892
Heart mass, g	1.58 ± 0.12	1.71 ± 0.15	0.055
Tibial length, mm	43.7 ± 1.1	43.9 ± 0.69	0.736
LV thickness, mm	3.27 ± 0.19	3.59 ± 0.13	0.003*
RV thickness, mm	1.39 ± 0.13	1.49 ± 0.13	0.235
Heart mass/body mass, %	0.32 ± 0.03	0.36 ± 0.01	0.002*
Heart mass/tibial length, g/mm	0.036 ± 0.001	0.039 ± 0.003	0.065
LV thickness/tibial length	0.075 ± 0.004	0.082 ± 0.003	0.003*
Fasted blood glucose, mmol/L	6.1 ± 0.2	27.8 ± 1.2	<0.001*
Citrate synthase activity, µmol/min/(mg protein)	1.18 ± 0.26	1.25 ± 0.3	0.640

*Note*: Values are means ± SD. * indicates *p*‐value < 0.05.

Abbreviations: CON, control; LV, left ventricular; RV, right ventricular; T2D, type 2 diabetes.

### 3.2 Blood biomarkers

Table 2 presents circulating plasma metabolic markers associated with metabolism, inflammation, cardiac health, liver function and kidney function. The T2D group showed significantly higher plasma levels of urea (*P* = 0.048), low‐density lipoprotein (*P* = 0.031) and triglycerides (*P* = 0.001) than the control group. Other markers, including high‐density lipoprotein, lactate dehydrogenase, C‐reactive protein, albumin and creatinine, were not different between groups.

**TABLE 2:** Plasma biomarkers at the end of the experiment.

Parameter	CON (*n* = 9)	T2D (*n* = 6)	*P*‐value
Lactate dehydrogenase, U/L	408 ± 169.9	377.2 ± 108.5	0.675
C‐Reactive protein, mg/L	0.27 ± 0.05	0.26 ± 0.05	0.494
Albumin, g/L	35.2 ± 1.3	34.8 ± 1.1	0.433
Urea, mmol/L	5.8 ± 0.7	7 ± 1.1	0.048*
High‐density lipoprotein, mmol/L	1.54 ± 0.18	1.63 ± 0.2	0.377
Low‐density lipoprotein, mmol/L	0.44 ± 0.08	0.71 ± 0.23	0.031*
Alanine transaminase, U/L	53.3 ± 8.96	89.9 ± 39.8	0.075
Aspartate aminotransferase, U/L	137.6 ± 31.4	138 ± 46.2	0.987
Creatinine, µmol/L	23.4 ± 1.74	24.3 ± 1.8	0.355
Uric acid, µmol/L	20 ± 8.7	13 ± 6.1	0.118
Triglycerides, mmol/L	0.44 ± 0.11	3.23 ± 1.1	0.001*

*Note*: Values are means ± SD. Student's unpaired *t*‐tests were used to detect differences between groups. * indicates *p*‐value < 0.05.

Abbreviations: CON, control; T2D, type 2 diabetes.

### 3.3 Mitochondrial respiration

**FIGURE 2:**
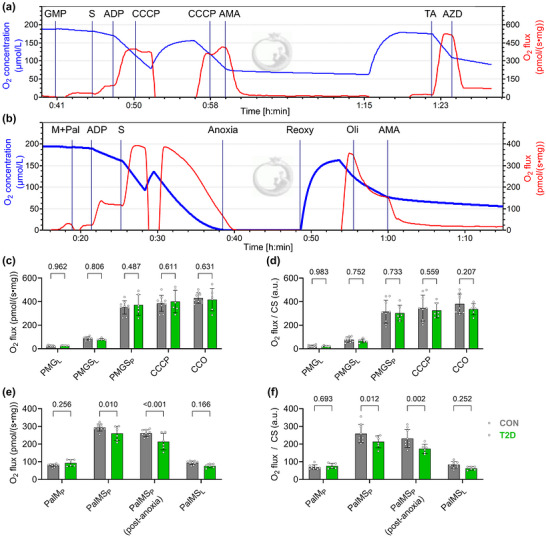
Mitochondrial respiration was supported by carbohydrate‐ and fatty acid‐derived substrates. Representative traces showing O_2_ concentration (blue) and O_2_ flux (red) across sequential respiratory states from titration protocol 1 (a) and Protocol 2 (b). Averaged O_2_ flux per tissue mass (c) or per citrate synthase activity (d) supported from carbohydrate‐based substrates. (e,f) Averaged O_2_ flux per tissue mass (e) or per citrate synthase activity (f) supported from fatty acid‐based substrates. CON group, *n* = 9, grey; T2D group, *n* = 6, green. Data are presented as the mean ± SD. Statistical analyses were performed using two‐way ANOVA with Fisher's least significant difference *post hoc* testing. Abbreviations: ADP, adenosine diphosphate; AMA, antimycin A; AZD, azide; CCCP, carbonyl cyanide *m*‐chlorophenyl hydrazone; CCO, cytochrome *c* oxidase; CON, control; G, glutamate; M, malate; P, pyruvate; PalM_p_, palmitate‐linked oxidative phosphorylation; PalMS_L_, palmitate and CII‐linked leak; PalMS_p_, palmitate and CII‐linked oxidative phosphorylation; PMG_L_, NADH‐linked leak; PMGS_L_, NADH‐ and CII‐linked leak; PMGS_P_, NADH‐ and CII‐linked oxidative phosphorylation; S, succinate; TA, TMPD and ascorbate; T2D, type 2 diabetes.

CS activity normalised to protein content, an indicator of mitochondrial content, was not significantly different between groups (*P *= 0.640; Table 1). Two substrate–uncoupler–inhibitor titration protocols were used to evaluate mitochondrial respiratory capacity supported from either carbohydrate‐based or fatty acid‐based substrates. In titration protocol 1, I measured O_2_ flux, ATP production, H_2_O_2_ production and mitochondrial membrane potential (Figure 2a). Titration protocol 2 measured O_2_ flux and ATP production (Figure 2b).

Leak respiration rate (PMGS_L_), using complex I (CI) substrates (glutamate, malate and pyruvate) and CII (succinate), reflects O_2_ consumption to counteract ion leaks in the absence of ATP production, which is facilitated by electron transport through the NADH‐linked and CII‐linked pathways. In the presence of saturating ADP, oxidative phosphorylation respiration (PMGS_P_) increases significantly, as a measure of O_2_ flux coupled to ATP synthesis. CCCP dissipates the proton gradient, preventing proton accumulation in the intermembrane space and thereby driving maximal electron transfer capacity through uncoupled respiration. Maximal electron transfer to CIV or cytochrome *c* oxidase (CCO) activity was measured using coupled ascorbate and TMPD, in which reduced TMPD donates electrons directly to cytochrome *c*, which are subsequently transferred to CIV, then to O_2_.

Mitochondrial O_2_ flux supported from carbohydrate‐based substrates across all respirometry states was not significantly different between groups when normalised to tissue mass (*P* = 0.487; Figure 2c) or CS activity (*P* = 0.733; Figure 2d). When supported with a fatty acid‐based substrate (palmitoyl‐l‐carnitine) and succinate stimulation, the T2D group had significantly lower mitochondrial O_2_ flux in OXPHOS respiration state (PalMS_P_) compared with the control group, normalised to either tissue mass (*P* = 0.010; Figure 2e) or CS activity (*P* < 0.001; Figure 2f).

### 3.4 Mitochondrial membrane potential, ATP flux and H_2_O_2_ flux measurements

**FIGURE 3:**
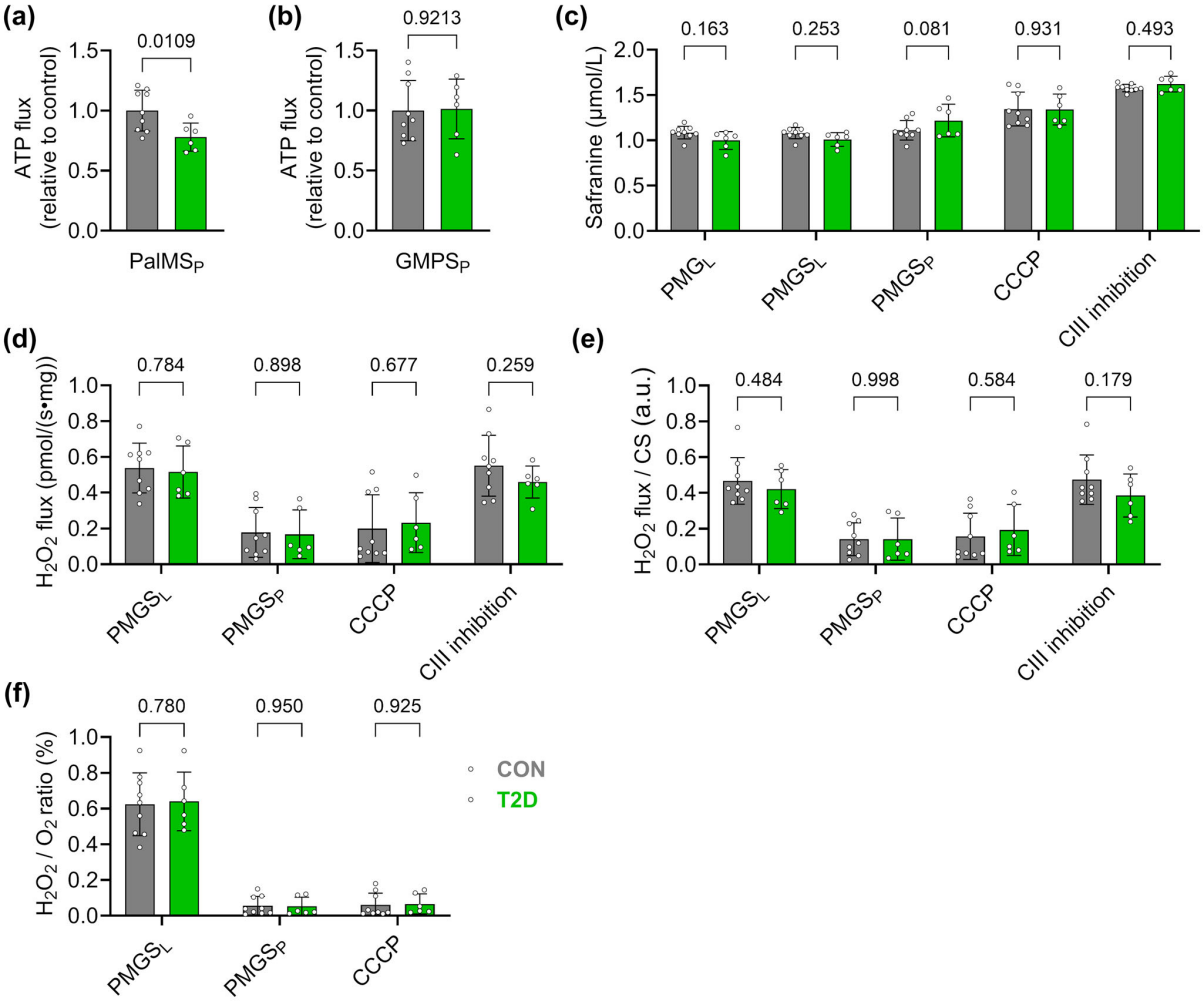
ATP production, membrane potential and H_2_O_2_ emission. Fluorometric assessment of mitochondrial function in left ventricular homogenates. Normalised ATP flux supported from fatty acid‐based substrates (a) or carbohydrate‐based substrates (b). (c) Mitochondrial membrane potential safranine dye signal at various respiratory states. H_2_O_2_ flux normalised per tissue mass (d), per citrate synthase activity (e) or relative to O_2_ flux (%) (f). CON group, *n* = 9, grey; T2D group, *n* = 6, green. Data are presented as the mean ± SD. Statistical analyses were performed using two‐way ANOVA with Fisher's least significant difference *post hoc* testing. Abbreviations: CCCP, carbonyl cyanide *m*‐chlorophenyl hydrazone; CON, control; PalMS_p_, palmitate and CII‐linked oxidative phosphorylation; PMG_L_, NADH‐linked leak; PMGS_L_, NADH‐ and CII‐linked leak; PMGS_P_, NADH‐ and CII‐linked oxidative phosphorylation; T2D, type 2 diabetes.

When supported with a fatty acid‐based substrate, the steady‐state OXPHOS ATP production rate or flux (PalMS_P_) was significantly lower in the T2D group than in the control group (*P* = 0.011; Figure 3a). The ATP production rate (GMPS_P_) did not differ between groups when mitochondria were supplied with carbohydrate‐based substrates (*P* = 0.921; Figure 3b).

Both mitochondrial membrane potential and H_2_O_2_ production measurements were performed with the support of carbohydrate‐based substrates. Mitochondrial membrane potential was not different between groups at all respiratory states (*P* > 0.16; Figure 3c). When normalised to tissue mass or CS activity or percentage of oxygen flux (H_2_O_2_/O_2_), H_2_O_2_ production rate at various respiratory states did not differ between control and T2D groups (Figures 3d–f).

### 3.5 Mass spectrometry metabolite profile

**FIGURE 4:**
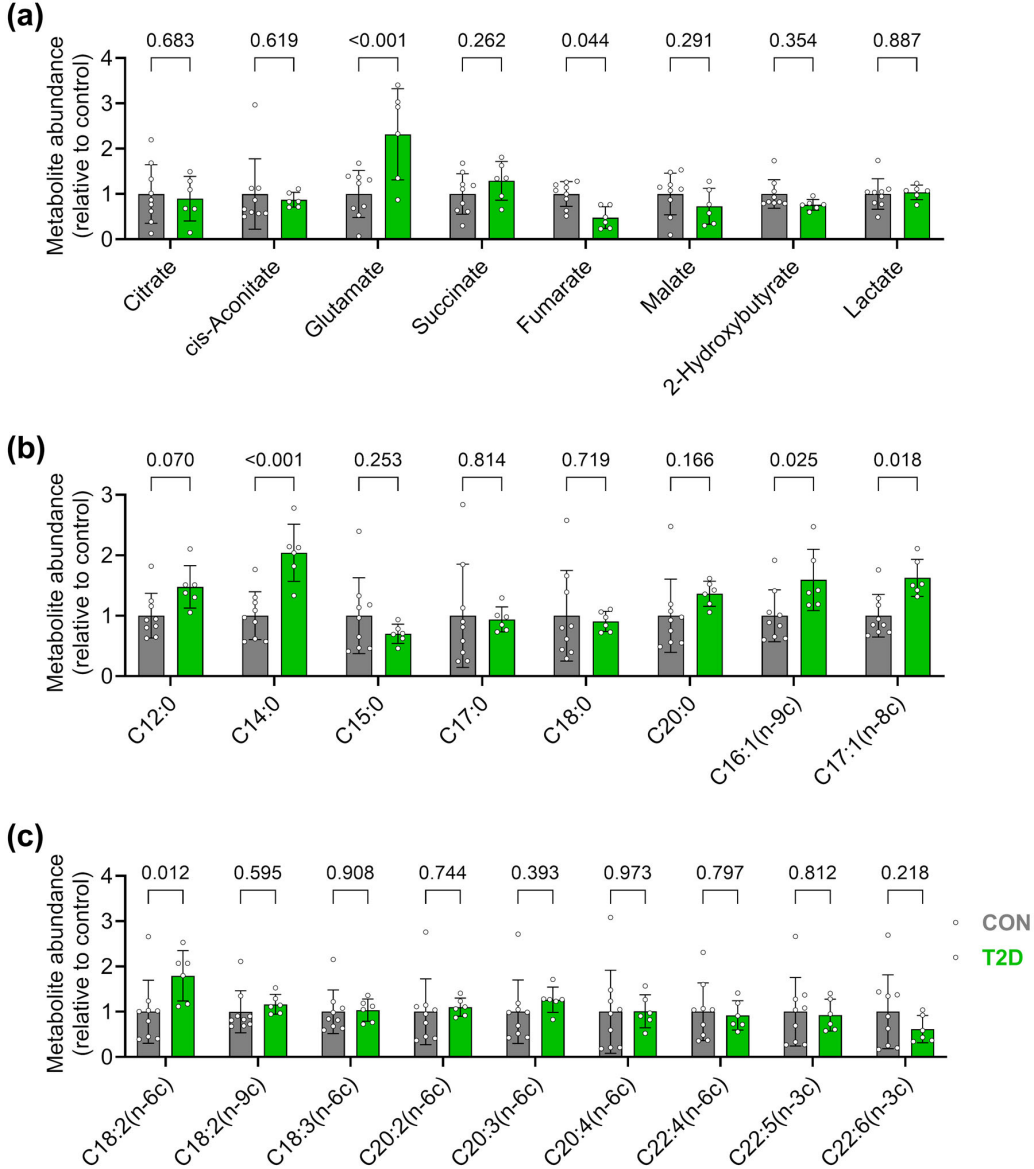
Myocardial metabolomic profile. Targeted gas chromatography–mass spectrometry analysis revealed increased abundance of several fatty acid species in T2D myocardium: Metabolites involved in the Krebs cycle, ketone bodies, and lactate (a), saturated and monounsaturated fatty acids (b), and polyunsaturated fatty acids (c). CON group, *n* = 9, grey; T2D group, *n* = 6, green. Data are presented as the mean ± SD. Statistical analyses were performed using two‐way ANOVA with Fisher's least significant difference *post hoc* testing. Abbreviations: CON, control; T2D, type 2 diabetes.

Gas chromatography–mass spectrometry metabolomic analysis of left ventricular tissues revealed changes in several metabolites (Figure 4). Key metabolites involved in the mitochondrial tricarboxylic acid cycle (TCA), including citrate, *cis*‐aconitate, succinate and malate, showed no difference between groups, except that fumarate was found to be lower in the T2D group (*P* = 0.044; Figure 4a). Glutamate, which links to the TCA cycle primarily by being converted into α‐ketoglutarate (a direct TCA intermediate), was found to be higher in the T2D group (*P* < 0.001).

Saturated fatty acids include C12:0, C14:0, C15:0, C17:0, C18:0 and C20:0. Monounsaturated fatty acids include C16:1 and C17:1. Polyunsaturated fatty acids include C18:2(n‐6c), C18:2(n‐9c), C18:3(n‐6c), C20:2(n‐6), C20:3(n‐6), C20:4(n‐6c), C22:4(n‐6c), C22:5(n‐3c) and C22:6(n‐3c). Figure 4b presents the metabolite abundance of saturated fatty acids and monounsaturated fatty acids, and Figure 4c presents the metabolite abundance of polyunsaturated fatty acids. Several fatty acid metabolites, including C14:0 (myristic acid; *P* < 0.001), C16:1(n‐9c) (palmitoleic acid; *P* = 0.025), C17:1(n‐8c) (*cis*‐8‐heptadecenoic acid; *P* = 0.018) and C18:2(n‐6c) (linoleic acid; *P* = 0.012), were found to be higher in the T2D group compared with the control group. The findings indicate that the diabetic heart accumulates fatty acids but cannot oxidise them efficiently.

## 4 DISCUSSION

This study provides new insight into how substrate availability shapes mitochondrial bioenergetics in the T2D heart, revealing that this effect emerges only when mitochondria are challenged with fatty acids. Carbohydrate‐supported respiration, ATP production, mitochondrial membrane potential and reactive oxygen species (ROS) emission were preserved, indicating that the core machinery of oxidative phosphorylation remains functional. Mitochondrial dysfunction occurs when mitochondria rely solely on fatty acids, which are the main substrates in the diabetic metabolic environment. The integrated metabolomic approach supports the respirometry findings that fatty acid supply to the myocardium is chronically elevated, whereas mitochondrial fatty acid β‐oxidation capacity does not match this increased supply, leading to elevated lipid intermediates. Central carbon metabolism remains preserved, as shown by largely unchanged carbohydrate‐supported respiration and TCA intermediates. This mismatch between substrate availability and oxidative capacity is a hallmark of metabolic inflexibility, a defining feature of diabetic cardiomyopathy.

A central insight from this study is that mitochondrial dysfunction in the diabetic heart does not stem from reduced mitochondrial content but from a mismatch between substrate supply and oxidative capacity. CS activity was unchanged, consistent with previous findings in human T2D hearts (Anderson et al., 2011; Croston et al., 2014; Duicu et al., 2016; Montaigne et al., 2014) and animal models (Mansor et al., 2013; Pham et al., 2025). Fatty acid‐supported respiration and ATP production were markedly impaired, aligning with previous findings in human T2D individuals (Ljubkovic et al., 2019) and an animal diabetic model (Cortassa et al., 2020), revealing a functional deficit that becomes apparent only when mitochondria are challenged with lipid fuels. This is particularly striking given the clear accumulation of multiple fatty acid species in the myocardium, indicating chronic lipid oversupply (Bonen et al., 2009; Chabowski et al., 2008; Finck et al., 2002; Ljubkovic et al., 2019). The metabolomics analysis consistently revealed a clear pattern of increased myocardial fatty acid abundance, including elevated myristic acid (C14:0), palmitoleic acid (C16:1), *cis*‐8‐heptadecenoic acid (C17:1) and linoleic acid (C18:2). These long‐chain fatty acids and monounsaturated fatty acids are abundant in the heart and are the main substrates for the β‐oxidation pathway. Increased levels of palmitoleic acid have been shown to be positively correlated with heart failure risk (Djoussé et al., 2012).

The diabetic heart is exposed to more fatty acids than it can effectively oxidise, a hallmark of metabolic inflexibility. The preserved abundance of most TCA intermediates (Figure 4a) further confirms that the mitochondrial dysfunction lies upstream of the TCA cycle, probably at the level of fatty acid transport, β‐oxidation enzyme activity or electron transfer through electron transfer flavoprotein. Prior work shows that lipid overload increases mitochondrial ROS generation (Ye et al., 2004) and mitochondrial uncoupling protein expression (Boudina et al., 2007; Cole et al., 2011) and decreases the proton motive force and ATP synthesis capacities (Ye et al., 2004). In this study, although these parameters were not measured in fatty acid conditions here, the metabolomic results strongly support this mechanism. The increase in glutamate abundance might reflect compensatory amino acid catabolism or altered α‐ketoglutarate cycling, further supporting a shift in substrate handling. Together, these findings support a model in which chronic lipid excess overwhelms mitochondrial oxidative capacity, driving lipotoxic stress, metabolic inflexibility and, ultimately, energetic insufficiency in the T2D heart.

In the presence of carbohydrate‐derived substrates, across all respiratory states, mitochondrial OXPHOS O_2_ flux, when normalised to either tissue mass or CS activity, remains unchanged, aligning with previous findings in T2D hearts (Anderson et al., 2011; Ljubkovic et al., 2019; Montaigne et al., 2014). Findings in early‐stage T2D rat hearts showed decreased carbohydrate‐supported mitochondrial respiration (Pham et al., 2025), suggesting mitochondria‐specific metabolic responses at various stages of the disease.

Fluorimetry in this study allowed real‐time tracking of ATP production, H_2_O_2_ emission, mitochondrial membrane potential and oxygen flux across defined respiratory states. Because fluorescent probes can influence mitochondrial respiratory function, it was important to confirm that the probes used here did not compromise respiratory capacity. Previous work has shown that MgG does not inhibit oxidative phosphorylation, supporting its use for ATP measurements (Cardoso et al., 2021). Safranine, in contrast, can partly suppress NADH‐linked respiration, hence membrane‐potential experiments were performed separately from respiration‐only runs (Krumschnabel et al., 2014). For H_2_O_2_ detection, my preliminary tests confirmed that 25 µM Amplex UltraRed maintained linear calibration curves without affecting oxygen flux. Taken together, these controls ensured that the respirometry data reflected genuine mitochondrial function, and the ATP‐production protocol served as the primary reference for validating probe compatibility.

The findings of mitochondrial H_2_O_2_ production rate per O_2_ being between 0.1% and 3% are consistent with previous reports in healthy rat hearts (Fang et al., 2026), diabetic rat hearts (Pham et al., 2014), hypertensive failing rat hearts (Power et al., 2016) and human skeletal muscle (Pham et al., 2020). This leak state is characterised by high succinate and limited ADP, mimicking ischaemic conditions in vivo. In this state, H_2_O_2_ production increased rapidly to the highest level owing to reverse electron transport to form superoxide at CI (Murphy, 2009). Across all respiratory states, no difference in H_2_O_2_ production rate normalised to tissue mass, CS activity or per O_2_ consumed was found between groups, contrasting with reports of decreased mitochondrial H_2_O_2_ production rate in a T1D rat heart (Pham et al., 2014) or increased in T2D mice (Parker et al., 2021) or T2D patients (Anderson et al., 2011). Differences in the use of diabetic models might account for these discrepancies.

Likewise, mitochondrial membrane potential or ATP production in carbohydrate‐supported substrate conditions was not different between groups, suggesting that mitochondrial capacity for glucose oxidation is adaptively maintained when carbohydrate metabolite abundance remains unchanged in this T2D rat model. The measurement of ATP production rate has been well characterised in left ventricular tissues from animal studies when glucose‐derived substrates support mitochondrial respiration. Animal studies have reported decreased ATP production rate in T1D rat hearts (Pham et al., 2014) or in T2D mouse hearts (Boudina et al., 2007) or hypertrophied rat hearts (Power et al., 2016), but unchanged in the early stage of T2D rats (Pham et al., 2025). To my knowledge, this is the first study to report mitochondrial ATP production with two different fuel substrates in late‐stage T2D hearts.

In this study, I included rats that exhibited phenotypes characteristic of established or late‐stage T2D rat models. Clear evidence was shown by their slight weight loss after the STZ injection, after which body mass was maintained, despite the high‐fat diet fed throughout the study. The findings of the systemic and cardiac phenotypes, including poorer glucose homeostasis, cardiac hypertrophy and elevated low‐density lipoprotein and triglyceride levels in T2D rats, are consistent with a previous study showing the established or later stages of the T2D rat model (Guo et al., 2018; Mansor et al., 2013) and with clinical observation of increased left ventricular wall thickness in T2D human hearts (Devereux et al., 2000), suggesting that β‐cell function becomes compromised and no longer meets the increased insulin demand.

Several limitations should be acknowledged. This study was conducted exclusively in male rats, and sex‐specific differences in mitochondrial metabolism are increasingly recognised in T2D, suggesting that the findings of the study might not fully capture female‐specific adaptations. Measurements of ROS and mitochondrial membrane potential were not performed in fatty acid substrate conditions because only one respirometer was available, and mitochondrial viability declined during the extended protocols required for multiple fluorophores and different fuel substrates. Future work will address this gap by directly assessing ROS and mitochondrial membrane potential in both carbohydrate and fatty acid conditions, allowing clearer comparison with studies reporting increased ROS and uncoupling during fatty acid oxidation in diabetic heart tissue (Boudina et al., 2007; Ye et al., 2004). Cytochrome *c* was not added to test outer mitochondrial membrane integrity because its use interferes with fluorescence‐based measurements; this remains a technical constraint of combined fluorometry and respirometry measurements. Although metabolomics revealed clear differences in substrate abundance, dynamic flux analyses in whole‐heart preparations will be needed to determine how these substrates are used. Finally, the incorporation of echocardiographic assessment of cardiac function, as shown in similar rat models (Liu et al., 2016), would strengthen the link between mitochondrial energetics and whole‐organ performance.

## 5 CONCLUSION

In conclusion, the diabetic heart shows a clear substrate‐specific energetic defect. Its mitochondria can readily oxidise carbohydrate‐derived fuels but fail to use fatty acids efficiently, even when these lipids accumulate in excess. This mismatch between substrate supply and mitochondrial capacity captures the essence of metabolic inflexibility in T2D and highlights lipid overload as a key driver of mitochondrial inefficiency. By defining where this breakdown occurs, the present findings lay the groundwork for future studies aimed at improving lipid handling and restoring β‐oxidation capacity as a strategy to rebalance cardiac energetics in diabetes.

## AUTHOR CONTRIBUTIONS

Toan Pham designed the study, performed the experiments and was responsible for statistical data analysis, figure preparation and manuscript drafting. Toan Pham approved the final version of the manuscript and agrees to be accountable for all aspects of the work in ensuring that questions related to the accuracy or integrity of any part of the work are appropriately investigated and resolved. All persons designated as authors qualify for authorship, and all those who qualify for authorship are listed.

## CONFLICT OF INTEREST

None declared.

## Data Availability

The data that support the findings of this study will be made available from the corresponding author upon request.
